# Changes in Gene Expression of the MCU Complex Are Induced by Electrical Stimulation in Adult Skeletal Muscle

**DOI:** 10.3389/fphys.2020.601313

**Published:** 2021-01-26

**Authors:** Esteban R. Quezada, Alexis Díaz-Vegas, Enrique Jaimovich, Mariana Casas

**Affiliations:** Center for Exercise, Metabolism, and Cancer, Physiology and Biophysics Program, Biomedical Sciences Institute (ICBM), Faculty of Medicine, University of Chile, Santiago, Chile

**Keywords:** mitochondria, calcium handling, muscle plasticity, ATP release, IP3R

## Abstract

The slow calcium transient triggered by low-frequency electrical stimulation (ES) in adult muscle fibers and regulated by the extracellular ATP/IP3/IP3R pathway has been related to muscle plasticity. A regulation of muscular tropism associated with the MCU has also been described. However, the role of transient cytosolic calcium signals and signaling pathways related to muscle plasticity over the regulation of gene expression of the MCU complex (MCU, MICU1, MICU2, and EMRE) in adult skeletal muscle is completely unknown. In the present work, we show that 270 0.3-ms-long pulses at 20-Hz ES (and not at 90 Hz) transiently decreased the mRNA levels of the MCU complex in mice flexor digitorum brevis isolated muscle fibers. Importantly, when ATP released after 20-Hz ES is hydrolyzed by the enzyme apyrase, the repressor effect of 20 Hz on mRNA levels of the MCU complex is lost. Accordingly, the exposure of muscle fibers to 30 μM exogenous ATP produces the same effect as 20-Hz ES. Moreover, the use of apyrase in resting conditions (without ES) increased mRNA levels of MCU, pointing out the importance of extracellular ATP concentration over MCU mRNA levels. The use of xestospongin B (inhibitor of IP3 receptors) also prevented the decrease of mRNA levels of MCU, MICU1, MICU2, and EMRE mediated by a low-frequency ES. Our results show that the MCU complex can be regulated by electrical stimuli in a frequency-dependent manner. The changes observed in mRNA levels may be related to changes in the mitochondria, associated with the phenotypic transition from a fast- to a slow-type muscle, according to the described effect of this stimulation frequency on muscle phenotype. The decrease in mRNA levels of the MCU complex by exogenous ATP and the increase in MCU levels when basal ATP is reduced with the enzyme apyrase indicate that extracellular ATP may be a regulator of the MCU complex. Moreover, our results suggest that this regulation is part of the axes linking low-frequency stimulation with ATP/IP3/IP3R.

## Introduction

Skeletal muscle can modify its phenotype to adapt to different external stimuli, such as disuse (Goldspink et al., 1986; Kneppers et al., 2019), hypoxia (Baresic et al., 2014; Nguyen et al., 2016), physical exercise (Yan et al., 2012; Joseph et al., 2016), among others (Gundersen, 2011), in a process known as muscle plasticity. According to this, different muscles of the body differ in their phenotype depending on the function they perform, expressing different isoforms of contractile proteins and metabolic enzymes. Accordingly, muscles (and, in a more general manner, motor units) have been broadly classified into three groups: slow-fatigue resistance, fast-fatigue resistance, and fast-fatigable. The model of muscle phenotype adaptation to different types of exercise is intimately linked to patterned electrical stimulation (ES) from the motoneurons innervating them. The first evidence involving frequency of ES in triggering molecular events associated with muscle plasticity was originated on *in vivo* models of cross-innervation, where a transition from fast to slow phenotype was observed in fast muscles innervated with α-motoneurons belonging to slow motor units presenting a low-frequency, long-lasting firing pattern (Eccles et al., 1962; Salmons and Vrbova, 1969). We have demonstrated that the inositol triphosphate receptor (IP_3_R) mediates the frequency-dependent induced changes (excitation–transcription coupling process) in gene expression involved in muscle plasticity of adult skeletal muscle. In particular, our results show that IP_3_R (and associated calcium signals) has a role in the activation of transcriptional programs associated with a slow phenotype that are activated at low frequencies of stimulation (Jorquera et al., 2013), partially emulating muscle changes induced by aerobic training (Klitgaard et al., 1990; Casas et al., 2010; Jorquera et al., 2013).

Excitation–transcription coupling is a process linking patterned depolarization of the muscle fibers with the activation of specific signaling pathways downstream. After a train of electrical stimuli, the process is triggered by the activation of the voltage-dependent L-type calcium channel Cav1.1 or dihydropyridine receptor (DHPR) (Jaimovich et al., 2000; Araya et al., 2003). At low frequencies of stimulation, DHPR activates the release of adenosine triphosphate (ATP), from the inside of the muscle fiber to the extracellular medium, through type 1 pannexin channels (Panx1). Extracellular ATP and its metabolites can thus act in an autocrine and paracrine manner, activating purinergic receptors, which, in turn, activate phosphatidylinositol 3-kinase (PI3K) and downstream pathway that favors the production of the second messenger 1,4,5 triphosphate (IP_3_) (Araya et al., 2003; Buvinic et al., 2009; Jorquera et al., 2013). Subsequently, IP_3_ binds to the membrane receptor of the sarcoplasmic reticulum (SR) and the nuclear envelope, causing Ca^2+^ release from the SR and the consequent increase in the cytosolic and nuclear Ca^2+^ concentration, which modulates the activity of several transcription factors to foster transcription (Carrasco et al., 2003).

During muscle contraction, the energy requirements of the muscle fiber are increased several times compared to rest (Weibel and Hoppeler, 2005). In muscle cells, mitochondria are the main source of ATP, and its function can be stimulated by various molecules, such as adenosine diphosphate (ADP), adenosine monophosphate (AMP), and Ca^2+^ (Lazarowski et al., 2003; Yi et al., 2011). In the mitochondrial matrix, different enzymes, such as isocitrate dehydrogenase and α-ketoglutarate dehydrogenase, have Ca^2+^ as a co-factor (Cortassa et al., 2003). Consequently, increases in the intramitochondrial concentration of Ca^2+^ increase the activity of these enzymes, increasing the speed of the Krebs cycle as well as the production of reduced compounds (NADH and FADH_2_) that feed the electron transport chain and ATP synthesis (Diaz-Vegas et al., 2019). Therefore, Ca^2+^ entry into the mitochondria is key to maintain the balance between the metabolic requirements and the synthesis of ATP in the skeletal muscle (Baughman et al., 2011; Tarasov et al., 2012; Diaz-Vegas et al., 2019).

The mitochondrial calcium uniporter (MCU) is a highly selective Ca^2+^ channel located in the inner mitochondrial membrane. MCU mediates an electrogenic Ca^2+^ influx from the intermembrane space to the mitochondrial matrix (Wan et al., 1989; O’Donnell et al., 1998; Carafoli, 2010). Furthermore, MCU is associated with different regulatory proteins that modulate their affinity for Ca^2+^ (Fan et al., 2020). MCU and its regulatory proteins are collectively known as the MCU complex, where MCU is the protein forming the pore of the channel (Chaudhuri et al., 2013; Shanmughapriya et al., 2015). The main components of the MCU complex expressed in adult skeletal muscle are MCU, essential MCU regulator (EMRE), mitochondrial Ca^2+^ uptake 1 (MICU1), and mitochondrial Ca^2+^ uptake 2 (MICU2) (Murgia and Rizzuto, 2015). Among these, the MICU1/MCU ratio appears to be particularly important for the regulation of mitochondrial Ca^2+^ uptake (Paillard et al., 2017). Increases in cytosolic Ca^2+^ levels generate an increase in mitochondrial Ca^2+^ through MCU. This process takes place after depolarization of the muscle fiber, where a high increase in intracellular Ca^2+^ concentration occurs. The consequent rise in mitochondrial Ca^2+^ depends on the activation of ryanodine receptor 1 (RyR1) (a fast component of Ca^2+^ release) as well as IP_3_R (a slow component of calcium release) (Diaz-Vegas et al., 2018).

There is evidence pointing to a possible role of MCU in muscle plasticity. Indeed, the silencing of MCU has been suggested to induce muscular atrophy, and its overexpression generates muscular hypertrophy in murine models (Mammucari et al., 2015), although these results are somehow controversial (Kwong et al., 2018). Also, 9 weeks of strength training or high-frequency EE (60 Hz) in humans induces hypertrophy and increases MCU protein levels in skeletal muscle (Zampieri et al., 2016). Interestingly, microarray analysis of muscles overexpressing MCU or underexpressing MCU showed differential changes in expression of genes related to sarcomere organization, calcium regulation, differentiation, and development. Notably, when MCU was overexpressed, an increase in expression of genes related to Ca^2+^ homeostasis was observed (Chemello et al., 2015).

Recently, the role of Ca^2+^ in the control of expression of MCU has been studied using the Ca^2+^ ionophore ionomycin to increase intracellular calcium concentration (Shanmughapriya et al., 2015). The results showed that CREB binds to the MCU promoter and alterations in cytosolic Ca^2+^ levels induced changes in MCU levels. Interestingly, these results suggest the existence of a crosstalk between cytosolic Ca^2+^ levels and the control of mitochondrial Ca^2+^ buffering capacity mechanisms (Shanmughapriya et al., 2015). Studies using more physiological stimuli are thus needed to further explore this mechanism. Furthermore, in hippocampal and cortical neurons, a reduction of MCU levels after increases in cytosolic Ca^2+^ through activation of NMDA receptor has been described (Qiu et al., 2013).

These works reveal the importance of understanding the role that transient changes in cytosolic Ca^2+^ levels (induced by a physiological stimulus) play in the regulation of gene expression of MCU, MICU1.1, MICU2, and EMRE in a tissue such as skeletal muscle where Ca^2+^ is a key factor. Such regulation could modulate the Ca^2+^ buffering efficiency of mitochondria, generating a physiological control loop of intracellular Ca^2+^ signals. In the case of adult skeletal muscle, a phenotypic change is observed under low-frequency ES, with modifications, among others, in the expression of oxidative related metabolic enzymes and an increase in mitochondria content (Hood et al., 1989; Putman et al., 2004; Petrie et al., 2015). The possible changes in mitochondrial proteins related to its Ca^2+^ buffering capacity is a subject that remains poorly studied until now.

In this work, we show a decrease in mRNA levels of MCU, MICU1, MICU2, and EMRE specifically after low-frequency ES. Moreover, the changes observed appear in line with an asymmetric distribution of some of these proteins between fast and slow phenotype muscle fibers.

## Results

### 20-Hz ES Produces a Decrease in mRNA Levels of the MCU Complex

**FIGURE 1:**
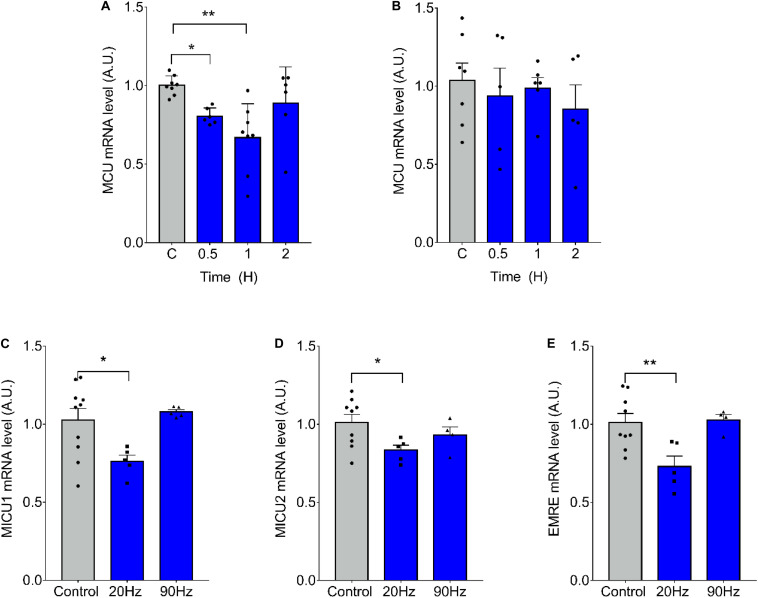
Low-frequency electrical stimulation (ES) transiently reduces mRNA levels of MCU complex in skeletal muscle fibers. Muscle fibers isolated from the *Flexor Digitorium Brevis* (*fdb*) muscle were stimulated with 270 pulses, 0.3 ms each **(A)** at 20 Hz (*n* = 6–8 per condition) or **(B)** 90 Hz (*n* = 5–7 per condition). mRNA levels of MCU decreased 1h post-ES **(A)**; while high-frequency stimulation produced no change **(B)**. The low frequency stimulation also decreased mRNA levels of MICU1 (*n* = 5–10 per condition) **(C)**, MICU2 (*n* = 4–9 per condition) **(D)** and EMRE (*n* = 4–9 per condition) **(E)** 1 h post ES. For **(A)** using the Kruskal–Wallis test with *post hoc* Dunn’s was applied. Values are presented as mean ± SEM. 18S was used as normalizer. *p < 0.05; **p < 0.01; ***p < 0.001.

We have previously demonstrated that ES of muscle fibers with 270 pulses, 0.3 ms long, at 20 Hz, induces changes in mRNA levels related to a slow-to-fast phenotypic transition, whereas the same amount of pulses at 90 Hz induces the inverse effect (Jorquera et al., 2013). We evaluated the effect of ES on mRNA levels of the MCU complex finding a significant decrease in MCU mRNA levels 30 min (1.00 ± 0.06 vs 0.81 ± 0.05 at 30 min) and 1 h (1.002 ± 0.061 vs 0.669 ± 0.216 at 1 h) after 20-Hz ES of isolated *fdb* muscle fibers (Figure 1A). High-frequency, 90-Hz ES did not produce changes in MCU mRNA levels (Figure 1B). Moreover, a decrease in the mRNA levels of MICU1 (1.03 ± 0.23; vs 0.761 ± 0.091 at 20 Hz), MICU2 (1.01 ± 0.16; vs 0.83 ± 0.07 at 20 Hz), and EMRE (1.01 ± 0.17 vs 0.73 ± 0.15 at 20 Hz) was also observed 1 h after 20-Hz ES, whereas 90-Hz ES did not generate any changes in mRNA levels of these genes (Figures 1C–E).

### Changes in mRNA Levels of the MCU Complex Are Dependent on Extracellular ATP and IP_3_R

**FIGURE 2:**
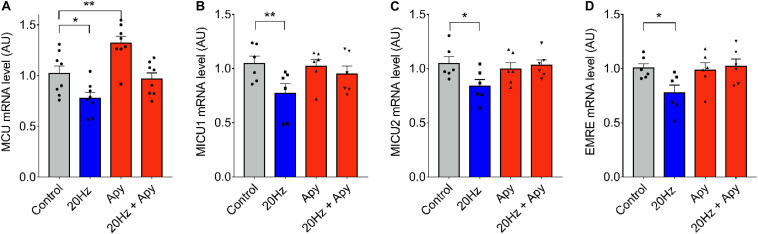
Low-frequency ES-dependent reduction in mRNA levels of MCU complex is dependent on extracellular ATP. Muscle fibers isolated from the *fdb* muscle were pre-incubated 30 min with apyrase (2 U/ml) before ES (as in Figure 1). The mRNA was extracted 1 h after the ES. MCU mRNA levels increased with pre-incubation with apyrase in the absence of ES **(A)**. Preincubation with apyrase prevented the low frequency-dependent reduction in mRNA levels of MCU (*n* = 8) **(A)**, MICU1 (*n* = 6) **(B)**, MICU2 (*n* = 6) **(C)**, and EMRE (*n* = 6) **(D)**. Values are presented as mean ± SEM. 18s was used as normalizer. *p < 0.05; **p < 0.01; ***p < 0.001.

To evaluate the downstream signaling after low-frequency ES, responsible for the observed changes in mRNA levels of MCU complex genes, we searched to determine the role of extracellular ATP. We have demonstrated that 20-Hz ES induces a release of ATP to the extracellular milieu trough Pannexin-1 channels (Jorquera et al., 2013). The ATP released acts over purinergic receptors to activate signaling cascades that activate, among others, the production of IP_3_ and the release of Ca^2+^ through IP_3_R, inducing changes in transcriptional activity of several genes (Casas et al., 2010). Figure 2 shows changes in MCU mRNA level in *fdb* muscle fibers pre-incubated with 2 U/ml of the ecto-nucleotidase apyrase to reduce extracellular ATP levels. The decrease observed in mRNA levels of MCU, MICU1, MICU2, and EMRE after a 20-Hz ES was absent when fibers were pre-incubated with apyrase (Figures 2A–E), when compared to control fibers. A significant increase in MCU mRNA levels (*C* = 1.02 ± 0.14; Apy = 1.32 ± 0.19) was also observed after incubation with apyrase (Figure 2A) in basal conditions (without ES). This effect of apyrase was not observed in MICU1, MICU2, and EMRE mRNA levels.

**FIGURE 3:**
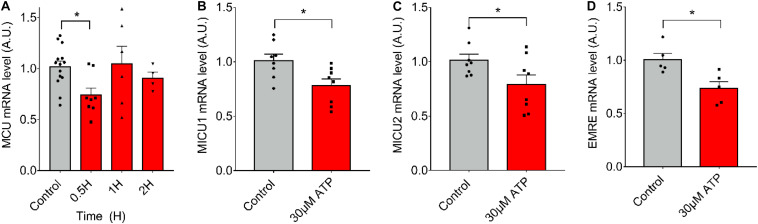
Extracellular ATP is sufficient to reduce mRNA levels of the MCU complex in skeletal muscle fibers. Muscle fibers *fdb* muscle and stimulated with exogenous ATP (30 μM). A decrease in mRNA levels of all the MCU complex components after 30 min of stimulation is observed. mRNA levels are shown normalized by mRNA level of control fibers. mRNA levels for MCU (*n* = 4–14) **(A)**, MICU1 (*n* = 8) **(B)**, MICU2 (*n* = 8) **(C)**, and EMRE (*n* = 5) **(D)** were measured. Values are presented as mean ± SEM. 18S was used as normalizer. *p < 0.05; **p < 0.01; ***p < 0.001.

To evaluate if ATP alone (in absence of ES) can induce the observed changes in mRNA levels after 20-Hz ES, we stimulated *fdb* muscle fibers with 30 μM of exogenous ATP and measured mRNA levels of the MCU complex. We observed that ATP exposure resulted in a significant decrease (*C* = 1.02 ± 0.20; 0.5 *h* = 0.74 ± 0.20) in mRNA levels of MCU after 30 min (Figure 3A). The same was observed at 30 min for mRNA levels of MICU1 (Control = 1.01 ± 0.17; 30 μM = 0.78 ± 0.17), MICU2 (Control = 1.02 ± 0.15; 30 μM = 0.79 ± 0.25), and EMRE (Control = 1.01 ± 0.13; 30 μM = 0.74 ± 0.15) after (Figures 3B–D). Similar effects were produced by extracellular ATP in an *ex vivo* model of complete *fdb* muscle (Supplementary Figure 1).

**FIGURE 4:**
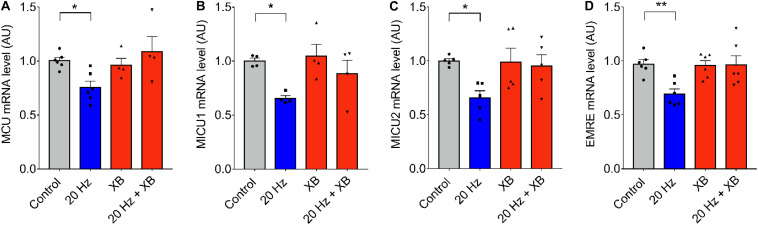
IP3R is involved in low-frequency ES-dependent reduction of mRNA levels of MCU complex. Muscle fibers *fdb* muscle were pretreated with the IP3R blocker, XB (10 μM) by 30 min before being electrically stimulated (as in Figure 1). The mRNA was extracted 1 h after electrical stimulation. Preincubation with XB prevented the low frequency-dependent reduction in mRNA levels of MCU (*n* = 6) **(A)**, MICU1 (*n* = 5) **(B)**, MICU2 (*n* = 5) **(C)**, and EMRE (*n* = 6) **(D)**. For **(C)** using the Kruskal–Wallis test with *post hoc* Dunn’s was applied. Values are presented as mean ± SEM. 18S was used as normalizer. *p < 0.05; **p < 0.01; ***p < 0.001.

As it was previously mentioned, signaling downstream ATP release and purinergic receptors activation can be mediated by the production of IP_3_ and Ca^2+^ released through IP_3_R. To test the role of IP_3_R, we blocked this intracellular Ca^2+^ channel using xestospongin B. No changes in MCU, MICU1, 0.MICU2, and EMRE mRNA levels after 20 Hz ES were observed when fibers were pre-incubated with 10 μM of xestospongin B (Figures 4A–D), suggesting a role of Ca^2+^ released through IP_3_R in the regulation of mRNA levels of the MCU complex.

### Mitochondria From Fast Muscles Have a Higher Content of MCU Complex Proteins

**FIGURE 5:**
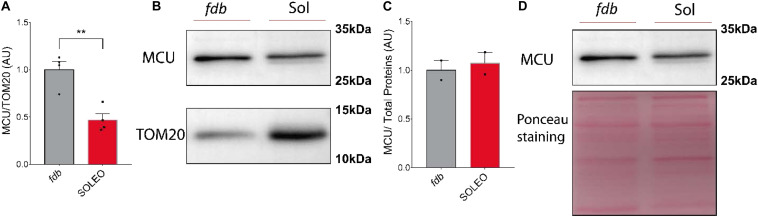
Mitochondria from *Flexor Digitorium Brevis* (*fdb*) muscle presented a higher content of MCU protein than soleus (sol) muscle. Representative Western Blot and quantification show the levels of the MCU protein in homogenates of *fdb* and Sol normalized by mitochondrial content using TOM20 as a normalizing protein (*n* = 4) **(A,B)**. When MCU content was normalized by total protein content, no differences between these two muscles were observed (*n* = 2) **(C,D)**. Values are presented as mean ± S.E.M. Values are presented as mean ± SEM. *p < 0.05; **p < 0.01; ***p < 0.001.

**FIGURE 6:**
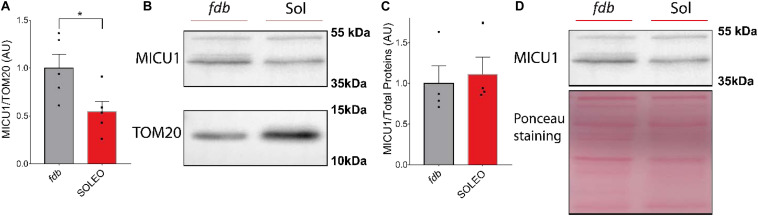
Mitochondria from *Flexor Digitorium Brevis* (*fdb*) muscle presented a higher content of MICU1 protein than mitochondria from soleus (sol) muscle. Representative Western Blot and quantification show the levels of the MICU1 protein in homogenates of *fdb* and sol, normalized by a mitochondrial marker (*n* = 5) **(A,B)** and total protein (*n* = 4) **(C,D)**. For **(C)** using the Mann-Whitney test was applied. Values are presented as mean ± SEM. *p < 0.05; **p < 0.01; ***p < 0.001.

We found a decrease in mRNA levels of the MCU complex after low-frequency ES. Considering that low-frequency ES is appropriate for slow-type motor units and that this type of ES can trigger transcriptional changes related to muscle fast-to-slow phenotype transitions, we hypothesized that proteins of the MCU complex could be differentially expressed in slow compared with fast phenotype adult muscles. We evaluated the levels of MCU and MICU1 proteins in a fast (*fdb*) and a slow (soleus) muscle by Western blot. We found a smaller amount of MCU (*fdb* = 1.00 ± 0.18; sol = 0.47 ± 0.13) (Figure 5A) and MICU1 (*fdb* = 1.00 ± 0.32; sol = 0.55 ± 0.24) (Figure 6A) in soleus muscle compared with *fdb* muscles. The protein content was normalized by TOM20, indicating that the relative amount of the MCU complex is lower in mitochondria from soleus muscle compared to *fdb*. This difference is not significant when normalized by total proteins (Figures 5C, 6C). This is probably due to the intrinsic difference in mitochondria content between these muscles, a difference that is compensated by the normalization of MCU protein content with a marker of mitochondria such as TOM20. MCU, MICU1, and TOM20 membranes are shown (Supplementary Figures 2, 3).

## Discussion

We have demonstrated that low-frequency ES results in a decrease in the mRNA levels of MCU, MICU1, MICU2, and EMRE, while high-frequency ES does not generate modifications. Our laboratory has described a fine-tuned mechanism that relates the decoding of the frequency of stimulation by Cav1.1, ATP release, IP_3_R activation, and transcription changes related to fast-to-slow muscle phenotype transition (Jorquera et al., 2013). Therefore, if there is a differential calcium management between different types of muscle fibers (Carroll et al., 1997), it is to be expected that genes that regulate mitochondrial calcium uptake could be regulated by different stimulation frequencies. In the present work, we showed that mRNA levels of MCU, MICU1, MICU2, and EMRE are also regulated in a frequency-dependent manner, being affected only by low-frequency ES. We have pooled the data from all controls and 20 Hz from the different experiments (Supplementary Figure 4). The observed changes suggest a new process related to the phenotypic transition of a fast to slow muscle fiber, in this case, related to mitochondrial proteins other than those related to oxidative metabolism, classically described before in the plasticity process from fast to slow muscle phenotype transition.

Our results showed that in fibers stimulated at 20 Hz after pre-incubation with apyrase, mRNA of the MCU complex did not decrease (as it does when they are only ES at 20 Hz), showing no significant statistical difference between control and 20 Hz + Apy conditions, indicating that the effect of 20-Hz ES in the decrease of MCU complex mRNAs is lost when fibers are pre-incubated with apyrase. The same is observed when fibers are pre-incubated with xestospongin B (IP_3_R blocker). No statistical difference was found between mRNA levels of the MCU complex from fibers electrically stimulated at 20 Hz compared to those electrically stimulated at 20 Hz and pre-incubated with apyrase or xestospongin B. However, these results, together with those showing an increase in mRNA levels of the MCU complex after stimulation with exogenous ATP, although not conclusive, are consistent with the idea that the transcriptional effects observed on MCU, MICU1, MICU2, and EMRE after low-frequency ES would be mediated by slow calcium transients activated by the extracellular ATP/IP_3_ production/IP_3_R signaling pathway (Buvinic et al., 2009; Jorquera et al., 2013). The role of mitochondrial Ca^2+^ uptake over cytosolic Ca^2+^ signals after muscle fiber depolarization has been shown previously to play a role in excitation–contraction coupling (Yi et al., 2011). Moreover, there is also evidence that protein levels of MCU are susceptible to change after exercise and to ES in humans (Zampieri et al., 2016). Interestingly, it has been postulated that protective synaptic activity is related to a decrease in MCU protein levels, which is lost in the presence of a CaM kinase inhibitor, suggesting a role of cytoplasmic Ca^2+^ in MCU regulation (Qiu et al., 2013). On the other hand, the reduction in mRNA levels of the MCU complex is observed at shorter times (30 min compared to 60 min for ES) when fibers are stimulated with exogenous ATP; this phenomenon could be related to the final concentration of ATP reached in the T-tubules and their affinity for purinergic receptors (Lazarowski et al., 2003) or the activation of these receptors without the process involving depolarization sensing by Cav1.1 and triggering of ATP release through pannexin-1 channels.

The results showing an increase in mRNA levels of MCU in the presence of apyrase, in the absence of further stimulus, suggest that basal levels of ATP present in the extracellular medium would be inhibiting or repressing the transcription of this gene.

Our data provide new information on the relative amount of MCU and MICU1 in muscle of different phenotypes, showing the existence of higher protein levels of MCU and MICU1 per mitochondria in *fdb* than those belonging to the soleus. Therefore, the decrease in mRNA of the MCU complex after low-frequency ES of isolated fibers of the *fdb* muscle would favor a lower level of the MCU and MICU1 per mitochondrion, such as that present in a slow muscle. This could be considered an early metabolic response to the phenotypic shift from fast to slow muscle fiber (Loucif et al., 2020). The gradual increase in the number of mitochondria, together with a decrease in MCU complex content in response to a low-frequency stimulus, will allow adapting mitochondrial Ca^2+^ homeostasis to finally reach that of a slow muscle. Besides, the higher levels of TOM20 in slow muscle compared to fast muscle are consistent with that described with other mitochondrial proteins, such as ATP synthase and succinate dehydrogenase (Schiaffino and Reggiani, 2011; Khodabukus and Baar, 2015). Moreover, the kinetics of Ca^2+^ entry to the mitochondria have also been reported to be different between different fiber types (Sembrowich et al., 1985; Picard et al., 2008). Interestingly, it has been described that MCU overexpression causes neuronal death (Granatiero et al., 2019). On the other hand, stimulation of cortical and hippocampal neurons results in a decrease in mRNA and protein levels of MCU (Qiu et al., 2013), which have been associated with a protective effect preventing mitochondrial Ca^2+^ overload, thus preventing cytochrome C from triggering cell death. Likewise, it has been observed that the decrease in MCU in a model of cells from colon cancer results in a resistance to apoptosis (Marchi et al., 2013; Nemani et al., 2018). It appears then that a fine regulation of the MCU protein complex is needed to balance protection and cell death after different stimuli. In this sense, it has been proposed that the concentration of mitochondrial Ca^2+^ necessary to exceed the threshold to trigger the opening of the mitochondrial permeability transition pore (mPTP) is lower in a slow fiber compared to a fast fiber (Picard et al., 2008, 2012). Therefore, since the muscle contraction of a slow phenotype fiber results in regular and prolonged elevations in cytosolic (and possibly mitochondrial) (Ca^2+^), we can hypothesize that low-frequency ES appropriate for slow-type muscles could induce a decrease in the MCU complex to regulate the entry of Ca^2+^ to the mitochondria and to prevent mPTP opening and thus protect the muscle fiber from triggering cell death. Even if muscle contraction in slow phenotype muscle relies strongly on mitochondrial production of ATP, which depends on mitochondrial Ca^2+^, the lower protein abundance of MCU and MICU1 per mitochondria in the soleus muscle could be compensated in the first place by the higher mitochondrial content in slow phenotype muscle and also by phenotypic adaptations of the slow muscle, such as an increase in sensitivity to Ca^2+^ by the enzymes isocitrate dehydrogenase and α-ketoglutarate dehydrogenase, maintaining the metabolism according to energy requirements (McCormack and Denton, 1989; Hurst et al., 2017). Not only has the protein level been described as a regulator of mitochondrial calcium uptake (Qiu et al., 2013), the stoichiometry of MCU/MICU1 (Paillard et al., 2017), mitochondrial endoplasmic reticulum interaction (Ainbinder et al., 2015), calcium release from the IP3R (Diaz-Vegas et al., 2018), and mitochondrial membrane potential (Diaz-Vegas et al., 2018) have also been described. Also, it has been described that an adaptive process of a cell is supported by a previous metabolic change, such as the case of metabolic changes associated with the activation of T-cell (Loucif et al., 2020). Exercise increases transcription factors of mitochondrial biogenesis and muscle hypertrophy in humans (Ruas et al., 2012). Deletion of PGC-1α (regulator of mitochondrial biogenesis) decreases the number and size of the mitochondria and the mass of the soleus and affects muscle performance (Leone et al., 2005). Thus, the changes observed at the level of the MCU complex in *fdb* could signify a prior process for the regulation of cytoplasmic Ca^2+^ signals mediated by the mitochondria, changing the regulation of genes related to contractility and favoring muscle plasticity. Therefore, future research is required to evaluate changes induced by ES in protein expression of the MCU complex and the consequences of a reduction of the MCU complex in the different functions of a skeletal muscle fiber, in the muscle plasticity process.

## Materials and Methods

### Animals

This study was carried out following the guidelines of the Bioethics Committee of the Faculty Medicine, University of Chile (FONDECYT #1151293). Eight- to 10-week-old male C57/BL6J mice were obtained from the Central Animal Facility of the Faculty of Medicine, University of Chile. Mice were kept in a room with controlled temperature in a light–dark cycle of 12 h and fed *ad libitum*.

### Adult FBD Fiber Isolation

The isolated adult muscle fibers were obtained from the *fdb* muscle by enzymatic digestion of the whole muscle with 450–500 units/ml of collagenase type II (Worthington) for 90 min, followed by mechanical dissociation with Pasteur pipettes of different diameters as described previously (Casas et al., 2010). Fibers were plated in ECM (Sigma)-covered 35-mm plates in culture medium [Dulbecco’s modified Eagle’s medium (DMEM), 10% horse serum, and 1% penicillin/streptomycin]. Fibers were used for analysis 20 h after seeding.

### Electrical Stimulation

The skeletal muscle fibers were electrically stimulated by field stimulation with a device consisting of parallel platinum wires with alternate polarity, as described previously (Casas et al., 2010). The protocol consisted of 270 pulses, 0.3 ms each at 20 Hz or 90 Hz (Jorquera et al., 2013).

### mRNA Isolation, cDNA, and Real-Time qPCR

Total mRNA was isolated from the muscle complete of the *fdb* by using TRIzol^®^ reagent (Invitrogen) according to the manufacturer’s protocol. The same extraction protocol was used to obtain total RNA from isolated fibers after ES. The cDNA was obtained by reverse transcription reaction of 1 μg of total RNA using random primer and polyDT primers. Real-time qPCR was performed according to the recommendations of EvaGreen^®^ qPCR Mix Plus (ROX) using the following primers: MCU-fw: 5′-GTGCGCCTGTTTGTAACTCA-3′ and MCU-rv: 5′-CAAGACTCGCTAAGCCCTTT-3′, MICU1.1-fw: 5′-CTTTG ATGGAAAGGAGTTCTGGC-3′ and MICU1.1-rv: 5′-CCTCCA TGTCTACCTCTCCGT-3′, MICU2-fw: 5′-TGGAGCACGACG GAGAGTAT-3′ and MICU2-rv: 5′-GCCAGCTTCTTGACCA GTGT-3′, EMRE-fw: 5′-AACTTCGCTGCTCTGCTTGA-3′ and EMRE-rv: 5′-TGAGGCTGAGGGCTTTCCTT-3′, 18 s. The design of primers was performed using the AmplifX program and validated by Primer-BLAST.

### Western Blot

The *fdb* and soleus samples were homogenized with an electric homogenizer (Fluko, Shanghai, China) in a lysis buffer containing 20 mM Tris–HCl (pH 7.5), 1% Triton X-100, 2 mM EDTA, 20 mM NaF, 1 mM Na_2_P_2_O_7_, 10% glycerol, 150 mM NaCl, 10 mM Na_3_VO_4_, 1 mM PMSF, and protease inhibitors (Complete^TM^, Roche Applied Science). Proteins were separated using SDS-PAGE and transferred to PVDF membranes. The following antibodies and their dilutions were used: MCU (1:2,000; HPA016480, Sigma), MICU1 (1:2,000; HPA037480, Sigma), and TOM20 (1:10,000; ab186735, ABCAM). The protein bands in the blots were visualized using a WESTAR Supernova detection kit (Cyanagen, Bologna, Italy), super-resolution, and, further, ChemiDoc^TM^ MP System (Bio-Rad, United States). The intensity of the bands was determined with ImageJ densitometry analysis.

### Statistical analysis

The results were expressed as mean ± standard error (±SEM). For the difference between data groups, the two-tailed paired *t* test was used. For comparison of more than two groups, the one-tailed one-way ANOVA was used followed by Dunnett’s multiple comparisons test. In cases where the data could not pass Levene’s equal variance test and Shapiro–Wilk test, Mann–Whitney test, and Kruskal–Wallis test with *post hoc* Dunn’s, a non-parametric test, as indicated in the figure legend, was applied. The level of significance was set at *p* < 0.05. All statistical analyses were performed in GraphPad Prism 7 and SPSS25.

## Data Availability Statement

The original contributions presented in the study are included in the article/Supplementary Material, further inquiries can be directed to the corresponding author/s.

## Ethics Statement

The animal study was reviewed and approved by Comité Institucional de Cuidado y Uso de Animales (CICUA).

## Author Contributions

EQ performed experiments, analyzed results, and contributed to write the manuscript. AD-V analyzed results and contributed to design experiments. EJ planned experiments, discussed results, and contributed to wrote the manuscript. MC design the project, planned experiments, analyzed and discussed results, and wrote the manuscript. All authors contributed to the article and approved the submitted version.

## Conflict of Interest

The authors declare that the research was conducted in the absence of any commercial or financial relationships that could be construed as a potential conflict of interest.
