# Supplementary material for: Changes in Gene Expression of the MCU Complex Are Induced by Electrical Stimulation in Adult Skeletal Muscle
Source: Front Physiol. 2021 Jan 26;11:601313. doi: 10.3389/fphys.2020.601313 (PMC7870689; doi:10.3389/fphys.2020.601313)
Supplement: Supplementary file 1 [file Image_1.pdf]

APPENDIX Quezada et al, 2020

Supplementary Figures

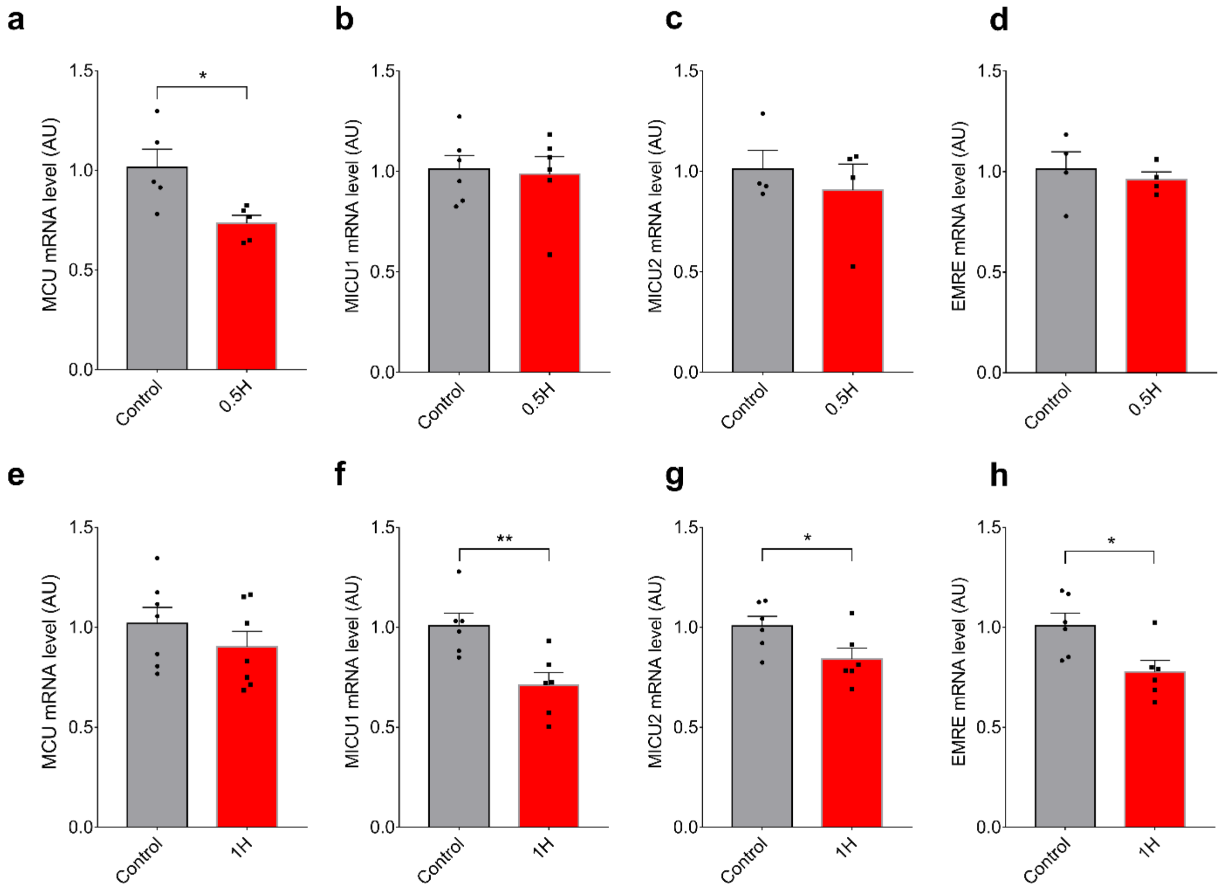

Supplementary figure 1. Extracellular ATP is enough to reduce mRNA level of the MCU in whole skeletal muscle. The complete muscle of the *fdb* was extracted and fixed in a chamber with Sylgard using needles. Enzymatic digestion was performed by collagenase IV for 40 min and a rest period of 1 hour. They were then pre-incubated with extracellular ATP (30 μM). The mRNA was extracted at 30 min **(a-d)** and 1H **(e-h)** post-stimulation. A decrease in MCU was observed at 30 min post-stimulation **(a).** A decrease in MICU1, MICU2, and EMRE were observed at 1h post-stimulation **(f-g)**. Values are presented as mean ± S.E.M. (n = 4-6). P0 was used as a normalizer. *, $p < 0.05$; **, $p < 0.01$; ***, $p < 0.001$.

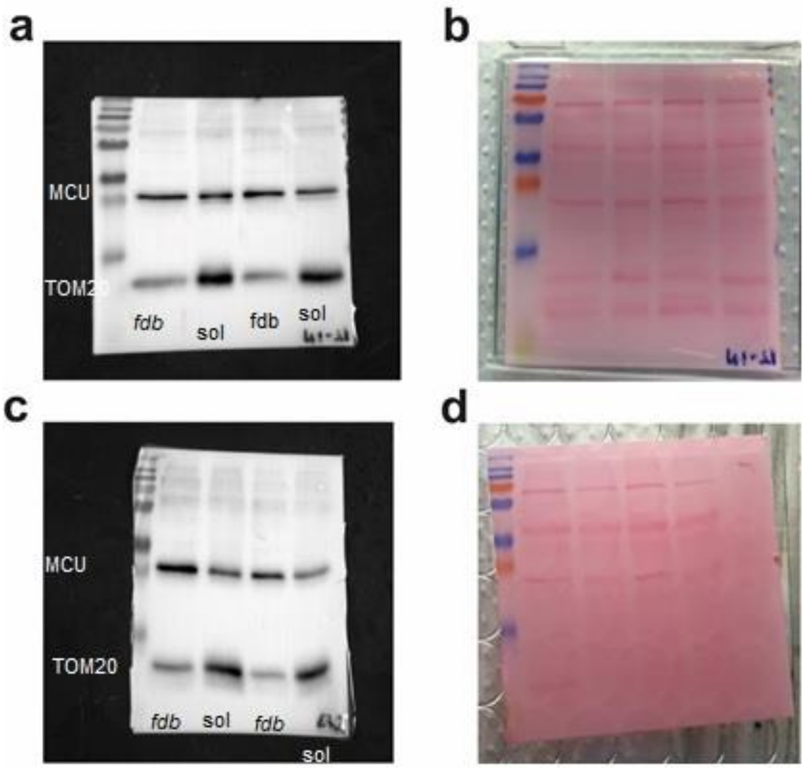

Supplementary figure 2. MCU and TOM20 bands on PVDF membranes. In a) and c) the MCU and TOM20 band (n = 4) is observed. In b) and d) the membranes are observed with Ponceau red (n=4). The samples were taken from complete muscles of the fdb and soleus.

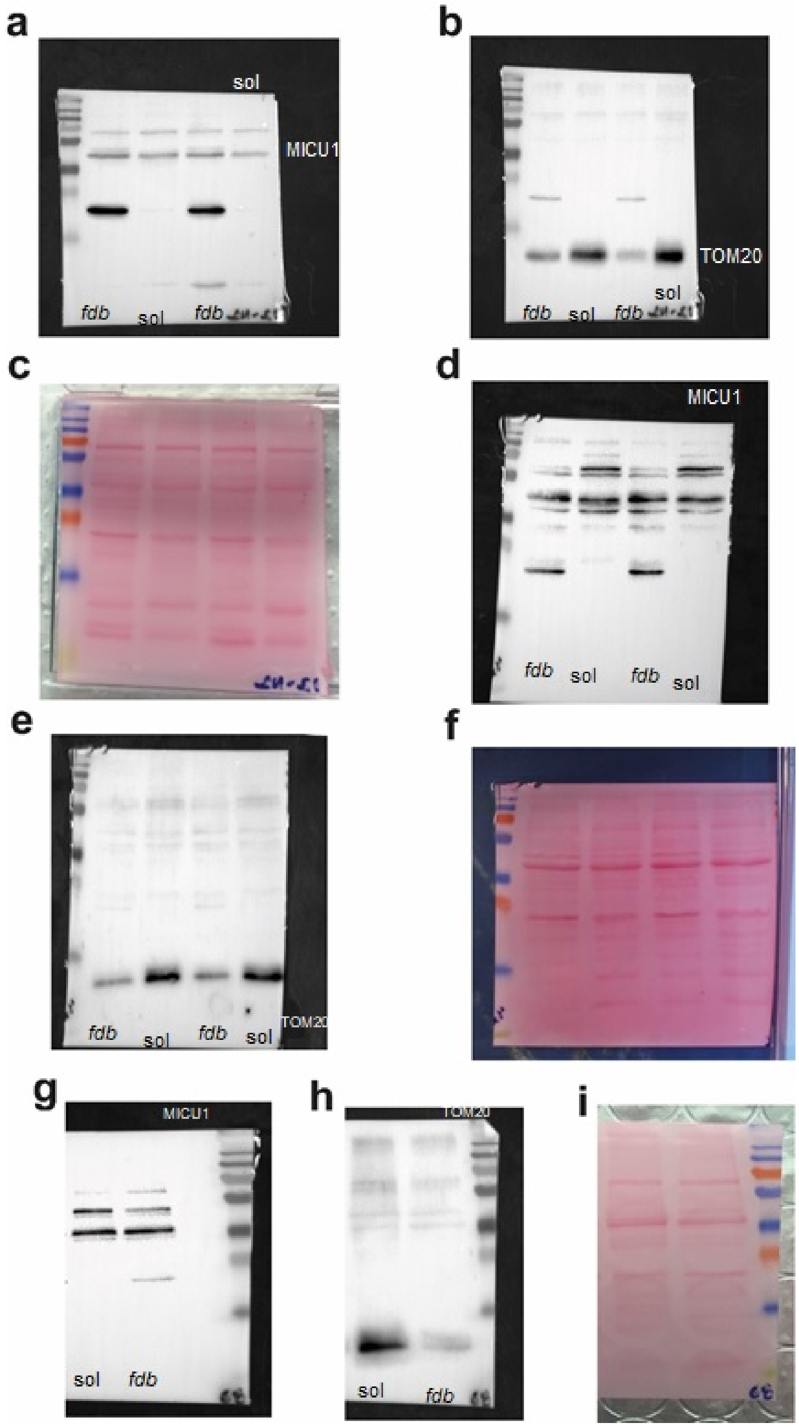

Supplementary figure 3. MICU1 and TOM20 bands on PVDF membranes. In a), d) and g) the MICU band is observed in the membranes (n = 5). In b), e) and h) the TOM20 band is observed in the membranes (n = 5). In c), f) and i) the membranes are observed with Ponceau red (n = 5). The samples were taken from the complete muscles of the *fdb* and soleus.

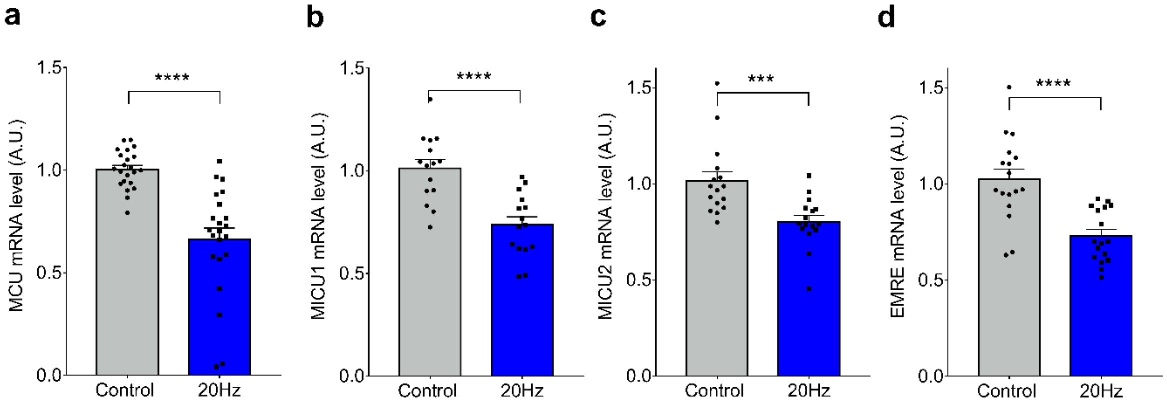

Supplementary figure 4. Pooled data indicates that low-frequency electrical stimulation (ES) transiently reduces mRNA levels of MCU complex in skeletal muscle fibers. Muscle fibers isolated from the *Flexor Digitorium Brevis* (*fdb*) muscle were stimulated with 270 pulses, 0.3 ms each **(a-d)** at 20 Hz. mRNA levels of MCU (n=22), MICU1 (n=15), MICU2 (n=16) and EMRE (n=17) decreased 1h post ES. The plates with isolated *fdb* fibers for each condition come from the same animal. For **(a)** and **(b)** using the Mann-Whitney test was applied. Values are presented as mean ± S.E.M. (n = 4-6). 18s was used as a normalizer. *, $p < 0.05$; **, $p < 0.01$; ***, $p < 0.001$; ****, $p < 0.001$.
